# A culturally tailored community gardening approach to improving physical activity, fruit and vegetable consumption, and psychological health among African American women: A pre-post feasibility study

**DOI:** 10.34172/hpp.025.43243

**Published:** 2025-07-15

**Authors:** Imani Canton, Vanesu Jakachira, Dawn Blackman, Heather Rose, Susan Aguiñaga

**Affiliations:** ^1^Department of Kinesiology and Health, Faculty of Kinesiology and Health, University of Illinois Urbana-Champaign, Champaign, USA; ^2^Department of Psychology, Faculty of Psychology, University of Illinois Urbana-Champaign, Champaign, USA; ^3^Randolph Street Community Garden, Champaign, USA

**Keywords:** Community gardening, Culturally tailored, Health disparities, Physical activity, Psychological health

## Abstract

**Background:**

African American (AA) women participate in low levels of physical activity (PA), under consume fruits and vegetables (FV), and experience poor psychological health. Increasing evidence suggests community gardening as an approach to positively affect health. The purpose of this study was to examine the feasibility of an 8-week culturally tailored community gardening intervention among middle-aged AA women.

**Methods:**

Eleven AA women (45-64 years) participated in a single group pre-posttest feasibility study during the summer of 2023 in Champaign, Illinois. The intervention included a novel approach to cultural tailoring by embedding Black History Knowledge (BHK) within the context of community gardening. Feasibility was assessed through a postintervention survey. Device-assessed PA (Fitbit Charge 3), FV consumption (Veggie Meter®), self-report PA, food frequency questionnaire, and psychological health were assessed at baseline and postintervention. Wilcoxon signed rank tests examined changes in pre-post measures. Effect size estimates were calculated using *r*.

**Results:**

Participants increased device-assessed daily step count (median 880 steps per day increase; r=0.53; *P*=0.028) and FV consumption (median 82-unit increase; r=0.51; *P*=0.016). There was a moderate effect on device-assessed light PA (r=0.45) and a small effect on device-assessed total PA (r=0.29), and perceived stress (r=-0.25). Sixty-seven percent (6/9) of women indicated that they would recommend this program to others.

**Conclusion:**

A culturally tailored, community-gardening intervention may be a feasible approach to increase device-assessed PA and FV consumption and improve psychological health among AA women, but future studies should be adequately powered.

## Introduction

 African American (AA) women are disproportionately burdened by cardiometabolic diseases such as obesity, cardiovascular disease, and type 2 diabetes^1^ affecting them at a prevalence of 55%,^2^ 57%,^3^ and 13%,^4^ respectively. Additionally, they affect AA women at higher rates than non-Hispanic white and Hispanic women.^5^ Both physical activity (PA) and adequate fruit and vegetable (FV) consumption are important non-pharmacological approaches to reducing risk of developing cardiometabolic diseases,^6,7^ yet both remain low among AA women.^8,9^ Systemic factors that contribute to these disparities include but are not limited to redlining, which has caused residential segregation and has resulted in AA women living in areas with less access to a high-quality built environment for PA participation and grocery stores for FV purchases.^10,11^

 Psychological distress refers to non-specific symptoms of stress, anxiety, and depression and high levels of psychological distress are indicative of impaired mental health^12^; positive psychological well-being refers to positive thoughts and feelings such as purpose in life, optimism, and happiness and promotes resilience.^13^ Psychological well-being plays an important role in health, with high levels of psychological distress associated with increased mortality risk^14^ and positive psychological well-being associated with better cardiovascular health outcomes.^13^ Both PA and FV consumption have been established as non-pharmacological approaches to reducing psychological distress,^15-18^ and as an extension, improving overall health outcomes. Less is known on the relationships between positive psychological well-being and PA,^19^ while there have also been inconsistent findings regarding FV consumption and positive psychological well-being.^20^ There has been low representation of minoritized populations in mental health related research,^21^ despite evidence indicating that AA women experience higher chronicity in depression and anxiety when compared to non-Hispanic white women.^22,23^ Additionally, discrimination, juggling multiple demands from a variety of roles (i.e. spousal, parental, communal), financial^24^ and occupational stress^25^ are many stressors that uniquely impact AA women. Simons and colleagues^26^ found that chronic exposure to discrimination predicts inflammation, and as a result, predicts the number of chronic diseases among mid-life AA women. Even less research has been conducted on positive psychological well-being amongst AA women.^27^ As such, there is a need for the development and implementation of novel, evidence-based interventions to eliminate health disparities and to achieve health equity amongst AA women.

 Community gardening may be an effective and unique approach to increase PA and FV consumption and improve psychological health amongst AA women. Qualitative and cross-sectional studies show that compared to non-gardeners, gardeners report feeling an increased sense of community,^28,29^ perceived better social cohesion,^30,31^ higher engagement in regular PA,^32,33^ feelings of positive mental well-being,^33,34^ and adequate intake of FV.^35^ However, few studies have been conducted among AA women, and even fewer community gardening interventions have been designed for AA women.^36,37^ Community gardening may specifically appeal to AA women because it is a highly social activity, and social support is a primary predictor and motivator of PA participation among AA women.^1,38,39^ Furthermore, the overall lighter intensity of gardening may also better appeal to AA women, as qualitative studies demonstrate that AA women often report preferences for walking, dance, and yoga/stretching instead of higher-intensity, traditional exercise like going to the gym to run and lifting weights.^40,41^ Recent data has demonstrated the potential health benefits of participation in light PA^42-44^ and it has also been recommended to examinetotal daily PA, which includes both exercise and non-exercise activity throughout the day,^43^ especially among low-active populations, as the traditional focus on increases in moderate-to-vigorous PA may initially be too challenging among low active populations.^45^

 Interventions targeting minoritized populations are recommended to be culturally tailored because tailoring takes into consideration the social and contextual correlates that may contribute to PA behavior.^46^ Consequently, including a culturally, historically relevant educational component to a community gardening intervention may also contribute to the adherence of a community gardening intervention and thus improve outcomes. Therefore, the purpose of this study is to assess the feasibility of “Tending to Our Roots to Increase Our Wellness” (TRIOWell), an 8-week culturally tailored community gardening intervention to increase daily total PA levels, FV consumption, and psychological health among middle-aged AA women. We hypothesized that TRIOWell would be a feasible approach to increase daily total PA levels, FV consumption, and psychological health among middle-aged AA women.

## Materials and Methods

###  Study design

 This study was a one group pre-posttest feasibility study. It can be used with small sample sizes and is an effective design for preliminary studies.^47^

###  Recruitment

 Approximately one year prior to recruitment, research staff developed rapport with a local church, local gardeners, and park district directors. The team attended regular meetings with gardeners and directors, as well as attended church services and Bible study sessions. Recruitment took place between May and July 2023, and we aimed to recruit 15 women. Participants were recruited via in-person and media-based approaches, including churches, community events (i.e., local Juneteenth celebrations, local live music events), the University of Illinois Urbana-Champaign Black Faculty and Professionals Alliance newsletter announcement, The University of Illinois Urbana-Champaign e-Week, and word of mouth. Word of mouth and in person recruitment at local events were the most effective recruitment strategies. A major challenge was encouraging potential participants to leave their contact information with the research team member, as many people expressed interest in the study and were open to taking the flyer for themselves and to distribute to others but were less interested in leaving their personal contact information. To overcome this challenge, team members emphasized that it was a free program and explained that providing their name does not automatically sign them up for the program, but rather, it would allow them to be able to speak with a team member to receive specific details about the program that were not on the recruitment flyer. Those who were interested gave their contact information to a member of the research team and were contacted by the team to be screened for study eligibility.

###  Participants

 Participants were selected using snowball and purposive sampling to better reach an AA population, given the historical distrust that minoritized communities have toward researchers, and because we were targeting AA women. For the present study, we were seeking middle aged AA women as multiple chronic conditions emerge in this age group, and middle-aged adults are often omitted from research. In Champaign-Urbana, AA comprise about 18% of the population; however, few studies target this population. We were also seeking AA women who were low-active and with limited gardening experience to examine changes in PA. Additionally, participants also had to have access to a device that could pair with the Fitbit. Therefore, our inclusion criteria for the participants included: 1) Self-identify as AA and female, 2) 45-64 years, 3) not have gardened in the past two gardening seasons, 4) < 3 days or < 90 minutes of moderate-to-vigorous PA per week, and 5) access to a smartphone, laptop, and/or tablet. Exclusion criteria include: 1) use of walking aids (cane, walker, wheelchair) and 2) ineligibility according to the Exercise Assessment and Screening for You (EASY) assessment.^48^ Study staff conducted and obtained informed consent in-person during eligible participants’ baseline assessment appointments.

###  Testing

 The baseline assessments lasted for 1-hour in duration and consisted of questionnaires, demographics, vitals (i.e., resting heart rate and blood pressure), and anthropometric measures (weight and height). Participants also received their Fitbit Charge 3 and were provided with personalized e-mail addresses and passwords made by research assistants to create a Fitbit account to use the Fitbit app; the Fitbit app was downloaded to their cell phones during the baseline assessment (all participants had either iOS Apple or Android cell phone). They were provided with a brief orientation and instruction packet to learn how to use the Fitbit and the app. Participants were instructed to wear the Fitbit as soon as they received it during baseline assessment and for the duration of the study, including when not gardening (total of approximately 11 weeks). After participants completed their baseline assessments and received their Fitbits, they received $15. Participants also received $15 for completing post-intervention assessments. Additionally, participants who attended more than 80% of sessions were able to keep their Fitbits.

###  Demographics

 We collected demographic variables including, age, marital status, number of children, number of people in the household, caregiver status, educational attainment, and employment status. We also collected self-report health status including sleep time, smoking status, general health rating, and health history.

###  Intervention 

 TRIOWell was an 8-week culturally tailored, community gardening pre-post feasibility trial. It consisted of educational workshops and gardening. All research personnel were trained prior to the start of the intervention on how to use the study materials (i.e., Fitbit and Veggie Meter®). Research assistants practiced using the Veggie Meter® on each other and Fitbits were worn to learn how they track data and connect to the app. All intervention components were supervised by the TRIOWell study leader to ensure that each session began and ended at the same time and that all intervention components were carried out as written in the protocol. A description of the components is provided below.

####  Educational workshops

 TRIOWell consisted of a total of 15, twice-weekly, 30-minute educational workshops (group discussions) led by a research staff person. Educational workshops took place on the same day as gardening sessions and took place prior to the gardening sessions. One session during week 5 took place virtually on Zoom due to inclement weather. Educational workshops took place directly before the gardening sessions at two locations: a local church and library meeting room. Topics covered during the workshops included the history of gardening among AA women, addressing barriers to PA participation, environmental justice in Black communities, and climate change. The purpose of the workshops was to provide social support and complementary sessions to the gardening sessions via Black History Knowledge (BHK). Previous gardening studies demonstrate that adding an educational component to the gardening enhances the program^36^ and culturally-tailoring the sessions through BHK would also likely enhance the receptivity of the programming. The curriculum is predetermined, where each day there is a different lesson. We encouraged an open dialogue and discussion at each session, but many sessions included accompanying activities to assist in discussion development (i.e., listening to a podcast, worksheets). The full curriculum can be found in Table 1.

**Table 1:** Educational workshop curriculum

**Week**	**Topic**	**Theories/Models**
1.1	Getting to know each other Introduction to PA	Social cognitive theory-Knowledge
1.2	Addressing barriers to PA and utilizing PA assets What are some current barriers you face to being physically active? What is already in our surroundings that can help us become more active? How to set achievable PA goals?	Social cognitive theory- Self efficacy
2.1	Utilizing community as social support to engage in healthy behaviors Think of communities that you are a part of; list them out Do you feel that they support you in becoming physically active?	Social cognitive theory- Social support Socio-ecological model- Community level
2.2	All about community gardening Types of gardens When community gardening became popular What community gardens are used for	Socio-ecological model- Community level
3.1	History of AA women and gardening Which types of food did AA women grow? Who benefitted from their gardens?	Black history knowledge
3.2	Continued history of AA women and gardening Exploring AA women’s relationship with the environment	
4.1	Food justice/power Food access Exploring food and its relationship with the American Civil Rights Movement	Black history knowledge
4.2	Continued food justice/power How has food been used to resist white supremacy?	
5.1	Food systems What makes up a food system?	Socio-ecological model- Policy level
5.2	Food systems What programs/policies are there at the local, state, and/or federal level that address parts of the food system?	
6.1	Climate change and health What is climate change? How does it impact our health?	Socio-ecological model- Policy
6.2	Continued climate change and health Current programs and policies in place at the local/state/federal levels to combat climate change	
7.1	Environmental justice What is environmental justice? Current examples of environmental injustices	Black history knowledge Socio-ecological model- Policy
7.2	Environmental activism throughout Black history Historical activists, programs, and movements Current activists, programs, and movements today	Black history knowledge

*Week 8: The participants played a team style trivia game using questions from the education workshop topics. It was a timed competition, and the teams could use their notes. The team with the most correctly answered questions won a prize. On the final day of the program, we hosted a potluck at the church.

####  Gardening intervention

 Participants engaged in a total of 15, twice-weekly, 1-hour long gardening sessions led by a horticultural educator from the local community. Gardening sessions took place on the same day as educational workshops and took place directly after the educational workshops. One session during week 5 was rescheduled to the following week due to inclement weather; thus, participants engaged in 3 gardening sessions during week 6. The gardening sessions took place following the education workshops at two community gardens both located in Champaign, Illinois. Participants gardened at one garden site during the week and at the additional gardening site on the weekend. During the gardening sessions, the horticultural educator, who was a 39-year-old Black woman, followed a lesson plan, as well as reminded the women to keep moving as much as possible and to also take breaks as needed. Much of the learning was experiential, where the horticultural educator verbally instructed, while physically demonstrated gardening skills to the participants. In the beginning sessions, participants were instructed on how to prune and weed, given that these two tasks are central to gardening upkeep. Participants were often split into groups and tasked with different jobs to gain experience in a variety of gardening skills. For example, during the first session, participants pruned tomato bushes, while another group weeded the garden. The groups switched after 20 minutes. Given that we utilized existing community gardening spaces, participants also assisted with other gardeners’ work, including watering the plants, and harvesting the produce. The horticultural educator donated the harvested fruits and vegetables to the surrounding community and the participants often took home with them their harvested fruits and vegetables including but not limited to bell peppers, jalapeño peppers, pears, and tomatoes.

####  Weekly phone calls

 Participants received weekly phone calls, (but also received emails or texts as after the first week of calls, some women preferred email or text) as reminders for upcoming sessions, to troubleshoot Fitbit concerns, to give reminders to wear the Fitbit as much as possible, and to discuss general PA goals. We also included weekly communication as a form of frequent contact to show a strong sense of caring, which is a recruitment and retention strategy suggested by Staffileno and Coke^49^ when working with Black women participants. During conversations, some women were vulnerable (i.e., embarrassed that they only took a certain amount of steps) during the phone calls, for example, and in response, research staff responded with compassion and encouraged participants to continue to make changes no matter how small.

###  Framework and theoretical basis of the intervention

 The intervention was modeled after a theoretical model called, “Amplifying Health Through Community Gardens,” (AHTCG) which is designed to link community gardens and health.^37^ This theoretical model is informed by many theoretical frameworks, like socio-ecological models, social cognitive and social determinant theories, the relational nature between people and places, and social capital. Alaimo and colleagues describe community gardens as a “multi-component, behaviorally based socio-environmental intervention,” given that they can affect intrapersonal, interpersonal, and environmental processes, as well as influence health behavior changes, like diet and PA, which influence chronic diseases and mental health.^36,50^ TRIOWell intervention components primarily aligned with the Social Cognitive Theory and the Socioecological Model.

 Social Cognitive Theory (SCT)^51^ postulates that human behaviors result from the mutual and changing interactions between personal factors and socio-environmental factors. Socioecological model (SEM) provides a theoretical framework for understanding the interrelations among a variety of personal and environmental factors in human health and illness.^52^ The levels of influence on health behaviors in this framework may include intrapersonal factors, interpersonal interactions, organizational policies and resources, community and geographic resources, structures and systems, and policy factors.^46^ Box 1 provides brief explanations of how the intervention embedded constructs and concepts from these theories.

**Box 1. Amplifying health through community gardens (social cognitive theory and socio-ecological model) constructs and concepts that guided the intervention components**

**Intrapersonal Level**- involves individual attitudes, knowledge, beliefs, and perceptions that influence a behavior

- Behavioral capability: knowledge and skill to perform a PA
○ **Education workshop topics** addressed:
    ▪ Definitions of PA and exercise
    ▪ Different types of PA (i.e., leisure, transportation)
    ▪ Barriers and assets of PA
○ **Gardening instruction:**
    ▪ Horticultural educator led gardening sessions to teach participants how to garden, which is a type of leisure PA
- Self-efficacy: confidence in oneself to take action and overcome barriers
○ **Education workshop topics **addressed:
    ▪ Barriers and assets of PA topic
    ▪ Encourage engagement in all types of PA to demonstrate that small changes are enough
○ **Weekly phone calls**
    ▪ Brainstormed ways to overcome barriers to achieve any personal PA goals
- Self-regulation: ability to manage social, cognitive, and motivational processes to achieve a desired goal
○ **Fitbit Charge 3 and Fitbit app**
    ▪ Participants utilized the Fitbit and the app to self-monitor and track daily PA (i.e., steps, heart rate)
○ **Education workshops**
    ▪ Opportunity to gain social support
○ **Weekly phone calls**
    ▪ Outcome Expectations: anticipated outcomes of engaging in PA
○ **Education workshop topics**
    ▪ Outcomes of participating in community gardening and other types of PA
      - Better mental health
      - Hypertension management
      - Increased energy
      - Reduced risk of Type 2 diabetes

**Interpersonal Level**- social influence from friends and family and norms within social networks

- Social Support: the perception and actuality that you are cared for, have assistance from other people, and that you are a part of a social network
○ **Education workshops**
    ▪ Group-based discussions
    ▪ Topic on community as social support for PA
      - Definition of social support
      - Types of social support
      - Historical connection draws on the historical relevance of kinship in Black communities
○ **Gardening sessions**
    ▪ Communal gardening sessions with participants, horticultural educator, and research staff

**Community Level**- the influence of settings in which people have social relationships, like schools, workplaces, and neighborhoods

- **Utilization of established locations in communities where participants live**
  ○ Randolph Street Community Garden- Champaign, IL
  ○ Doulgass Park Community Garden- Champaign, IL
  ○ Champaign Public Library Douglass Park Branch- Champaign, IL
  ○ Champaign Church of the Brethren- Champaign, IL
- **Established rapport with staff at the Champaign Park District, volunteers with the Solidarity Gardens, and garden steward at the Randolph Street Community Garden to help promote gardening at their locations as a wellness program**

###  Cultural tailoring of TRIOWell

 Resnicow’s culturally-tailoring framework describes *surface* and *deep-structure* cultural adaptations when designing culturally-sensitive interventions.^53^ Surface structure cultural adaptations are the simplest forms of tailoring, where intervention materials are matched to “superficial” characteristics of the targeted population. Deep structure cultural adaptations are more complex and requires a deeper understanding of the cultural, social, historical, environmental, and psychological factors that influence health behaviors of the targeted population. Surface-level tailoring of TRIOWell included:

- Holding education workshops at a church, given 75% of AAs identify as Christian^54^
- Including images of Black women and families in the workshop materials
- Intervention delivery solely by Black women.

Deep-structure tailoring included:

- Fostering social support through group-based discussions during the education workshops since many AA women value close and kinship-like relationships in their lives
- Considering collectivism and highlighting during discussions how staying healthy themselves will allow them to better care for their family and communities
- Embedding BHK throughout the education workshops to foster racial pride

###  “Black History Knowledge” as a novel approach to culturally-tailoring PA interventions

 The BHK model is a framework that attempts to illustrate the interrelationships of risk associated with structural and systemic oppression, BHK awareness domains, stress and coping responses, and mental health outcomes because of these relationships. The model is intended to describe these relationships among Black youth, where evidence has indicated that Black youth who have a strong knowledge of their history may better cope with race-related stressors and discrimination.^55,56^ However, it also has implications for Black people across the lifespan. Research suggests that race-related stress that occurs during youth may have long-lasting psychological effects into adulthood page,^57^ so continuing to learn about the vast and unique history of Black people may also be beneficial for adult Black women. The model centers four BHK awareness domains: 1) awareness of structure of race and racism in the U.S., 2) awareness of contributions and achievements, 3) awareness of capital positioning (social, political, economic), and 4) awareness of cultural strengths that foster empowered action. These domains intend to emphasize strength, resilience, and achievements within Black history versus solely focusing on a deficit-based Black American historical education. We embedded and targeted these domains within the education workshops by including a “Historical Connection” with each topic. Examples of historical connections from the workshops are available in Supplementary file 1.

###  Outcomes

 Self-reported outcomes were assessed using Research Electronic Data Capture [REDCap]^58,59^ hosted at the University of Illinois Urbana Champaign, with a research personnel member reading the questionnaires to the participant and marking the responses for the participant. Questionnaires were filled out this way as another approach to build rapport with the participants.^49^

###  Physical activity outcomes

####  Device-assessed PA

 Participants were asked to wear the Fitbit Charge 3 on their non-dominant wrist for a minimum of 11 weeks in total: at least 2-weeks prior to the start of the intervention (Weeks -1 and 0), during the intervention (Weeks 1-8), and 1-week after the intervention (Week 9). Week -1 served as a trial week, as evidence indicates that participants may be reactive and increase their PA shortly after receiving a wearable^60^ and as such was not included in analysis. PA from Week 0 was included as baseline data. PA data are reported as steps, minutes in light-intensity PA, minutes in moderate-to-vigorous intensity PA, and total minutes in PA (light-intensity minutes + moderate-to-vigorous intensity minutes).

 Given that wear time data is not readily available through Fitbit’s Web API and a third party authorized platform was not used to assist with data collection, wear time had to be estimated. Using Balbim and colleagues’ Fitbit potential challenges and solutions as a guide,^61^ heart rate data was used as a wear time proxy because it can only be calculated if the Fitbit is worn. A valid day was at least 10 hours of heart rate data during waking hours. If less than 10 hours of heart rate data were present, this data was removed. Sometimes sleep data (Fitbit provides time asleep if worn during sleep) were available (wearing during sleep was not necessary for the study), so sleep data were also used to further validate PA time. It became evident that 500 steps per day and/or sedentary time at or around 1440 minutes were often associated with less than 10 hours of heart rate data, so it was decided to use 500 steps or less per day and/or at or around 1440 sedentary minutes as a benchmark of non-wear time, especially for those who did not have sleep data to validate against PA data. Data with those benchmark measurements were removed, too. Participants had to have at least 3 valid days of PA data to be used in analysis. Lastly, participants were taught how to sync their Fitbits, which included opening the Fitbit app every few days and refreshing the page.

####  Self-report PA

 The Recent Physical Activity Questionnaire (RPAQ),^62^ assesses time spent in usual PA in the past month in 4 domains (work, travel, recreation, and domestic life). Levels of PA energy expenditure, based off METs, can be calculated by multiplying the associated METs value by the number of hours performing the activity using this questionnaire. Self-reported total PA was calculated by summing domains of recreation PA (i.e., leisure) and work PA. This questionnaire has been validated to estimate total energy expenditure, PA energy expenditure, sedentary time, and time spent in vigorous PA in inactive men and women; it has also shown high reliability.

###  Fruit and vegetable consumption assessments

####  Device assessed FV consumption

 The Veggie Meter® is a portable device that measures skin carotenoid (colorful plant pigments found in fruits and vegetables with light filtering and antioxidant properties that deposit in the skin) concentrations. It uses reflection spectroscopy to assess reflection of light from the skin (fingertip) after exposure to a source of white LED light. The Veggie meter has been validated amongst adult samples and serum samples have been highly correlated with skin carotenoids that were measured using the Veggie meter. To use the Veggie meter, participants placed a clean and dry ring finger on their non-dominant hand on a convex lens where a white LED light is passed through while gentle pressure is applied to the fingertip. We took 3 separate measurements and calculated the average of the scores to use for analyses.

####  Self-reported food frequency questionnaire

 The MIND diet^63^ is a 15-item food frequency questionnaire that assesses the consumption of 10 brain healthy food groups (green leafy vegetables, other vegetables, nuts, berries, beans, whole grains, fish, poultry, olive oil and wine [moderate]) and 5 unhealthy food groups (red meats, butter/margarine, cheese, pastries/sweets, and fried/fast food). We assessed green leafy vegetables, other vegetables, and berries. A score of 0, 0.5, and 1 are given for each component according to the frequency of consumption. A score of 1 for an item indicates meeting the suggested intake of food item.

###  Psychological health 

####  Perceived stress

 We assessed perceived stress by using the Perceived Stress Scale-10 (PSS-10), a 10-item assessment^64^ designed to measure the degree to which an individual perceives and appraises life events as stressful.^65^ It is the most widely used psychological instrument for measuring the perception of stress and it is a valid and reliable shortened version of the original 14-item instrument. Questions ask about feelings and thoughts during the last month. In each question, respondents are asked how often they felt a certain way. Scores are summed across all scale items.

####  Anxiety

 We assessed anxiety using the Patient Reported Outcomes Measurement Information System (PROMIS) Short Form v1.0- Anxiety 8a.^66^ It is an 8-item self-report measure of anxiety symptoms experienced during the past 7 days. It contains a subset of anxiety items from the full PROMIS Anxiety item bank. Participants rate each item on a 5-point scale, indicating how often they experienced an item (1- *“Never” *to 5- *“Always”*). Raw scores were converted to t-scores based on published PROMIS scoring guidelines.^67^ For most PROMIS measures, a t-score of 50 is the average for the United States general population with a standard deviation of 10. For negatively worded concepts like anxiety, a t-score of 60 is one standard deviation worse than the general population and in contrast a t-score of 40 is one standard deviation better than the general population. Higher scores indicate experiencing anxiety symptoms often.

####  Depression

 We assessed depression PROMIS Short Form v1.0- Depression 8a.^66^ It is an 8-item self-report measure of depression symptoms experienced during the past 7 days. It contains a subset of depression items from the full PROMIS Depression item bank. Participants rate each item on a 5-point scale, indicating how often they experienced an item (1- *“Never” *to 5- “*Always”*). Raw scores were converted to t-scores based on published PROMIS scoring guidelines. Higher scores indicate experiencing depressive symptoms often.

####  Self-Efficacy

 We assessed self-efficacy by using the Barriers Specific Self-Efficacy Scale (BARSE)^68^ which measures the perception of an individual’s confidence to exercise 3 times per week for the next 3 months given potential barriers. For each item, participants indicated their confidence to complete that behavior on a 100-point percentage scale with 10-point increments, ranging from 0% (not at all confident) to 100% (highly confident). Scores are calculated by summing the confidence ratings and dividing by the total number of items in the scale, with a highest possible efficacy score of 100. BARSE has been validated amongst working women with high construct validity and high reliability (Cronbach’s alpha = 0.92).^69^

####  Meaning and purpose

 PROMIS Short Form v1.0-Meaning and Purpose 8a^70^ is an 8-item self-report measure of one’s sense of life having purpose and that there are good reasons for living. It contains a subset of meaning and purpose items from the full PROMIS Meaning and Purpose item bank. Participants rate each item on a 5-point scale, indicating to what extent a statement applies to their life (1-“*Not at all”* to 5-“*Very Much”*). Raw scores are converted t-scores based on published PROMIS scoring guidelines. For positively worded concepts like meaning and purpose, a t-score of 60 is one standard deviation better than the general population and in contrast a t-score of 40 is one standard deviation worse than the general population. Higher scores indicate hopefulness, optimism, goal-directedness, and feelings that one’s life is worthy.

####  General life satisfaction

 PROMIS Short Form v1.0 General Life Satisfaction 5a^70^ is a 5-item self-report measure that assesses cognitive evaluation of life experiences and whether one likes his/her life or not. It contains a subset of general life satisfaction items from the full PROMIS General Life Satisfaction item bank. Participants rate each item on a 7-point scale, indicating to what extent they agree or disagree with a statement (1- *“Strongly Disagree” *to 7- *“Strongly Agree”*). Raw scores are converted t-scores based on published PROMIS scoring guidelines. A higher score indicates better overall life satisfaction.

###  Data analysis

 Descriptive statistics (mean, frequency, percentage) were used to summarize demographic characteristics. Because of the small sample size (n = 11) and not meeting normality assumptions, we conducted non-parametric analyses. Wilcoxon signed-rank tests were used to examine pre- versus post-intervention changes in study outcomes, as this test does not require normality assumptions and is suitable for small, paired samples. Effect sizes were estimated by calculating, $r=\frac{Z}{\sqrt{N}}$ , where Z was the z-score obtained from the Wilcoxon signed-rank test and N is the number of observations. Calculating effect sizes is important for small sample sizes, as it provides a measure of the magnitude of the intervention’s effect. Statistical significance was set at a *P* value of < 0.5 as a point of reference because the study was not powered enough to detect significant changes in study outcomes.

 To ensure data quality control, research personnel checked all surveys and outcomes for missingness and contacted participants about missing data when necessary. Fitbits were synced to participant’s apps during their baseline testing to guarantee that they synced properly. If there was an issue with syncing, a new Fitbit was provided (e.g., one Fitbit did not sync and a different Fitbit was provided). Protocols for cleaning Fitbit data were provided to research assistants. Two research assistants were always present during any data entry to account for potential human error.

## Results

###  Demographics 

 Participants included in the analysis (n = 11) had a mean age of 50.8 (SD 6.4) and a mean BMI of 35.9 (SD 5.2) kg/m^2^. Overall, 36.8% (n = 4) were married, 27.3% (n = 3) were single, 27.3% (n = 3) were separated/divorced, and 9.1% (n = 1) were widowed. Additionally, 9.1% (n = 1) had a high school diploma/GED, 9.1% (n = 1) attended some college, 9.1.% (n = 1) had an Associate’s degree, 45.5% (n = 5) had a Bachelor’s degree, 9.1% (n = 1) had a Master’s degree, and 18.2% (n = 2) had a PhD. The participants were primarily full time employed (n = 7; 63.6%). Table 2 provides a description of participant characteristics.

**Table 2:** Participant characteristics

**Demographics**	**n**	**M±SD or %**
Age, years	11	50.8 ± 6.4
BMI, kg/m	11	35.9 ± 5.2
Blood pressure, mm Hg		
Systolic	10	114.8 ± 11.2
Diastolic	10	74.4 ± 5.6
Resting heart rate, bpm	10	82.3 ± 14.2
Education		
Bachelor's degree or higher	8	72.8
Employment status		
Full Time, ≥ 35 hours/week	7	63.6
Marital status		
Single or separated/divorced	6	54.6
Number of children		
3 or more	6	54.6
People in household		
4 or more	4	36.4
Caregiver (unpaid)	3	27.3
Sleep		
< 7 hours per night	6	54.6
General health rating		
Fair or good	9	81.9
Chronic condition		
High blood pressure	3	27.3

###  Feasibility 

####  Recruitment

**Figure 1:**
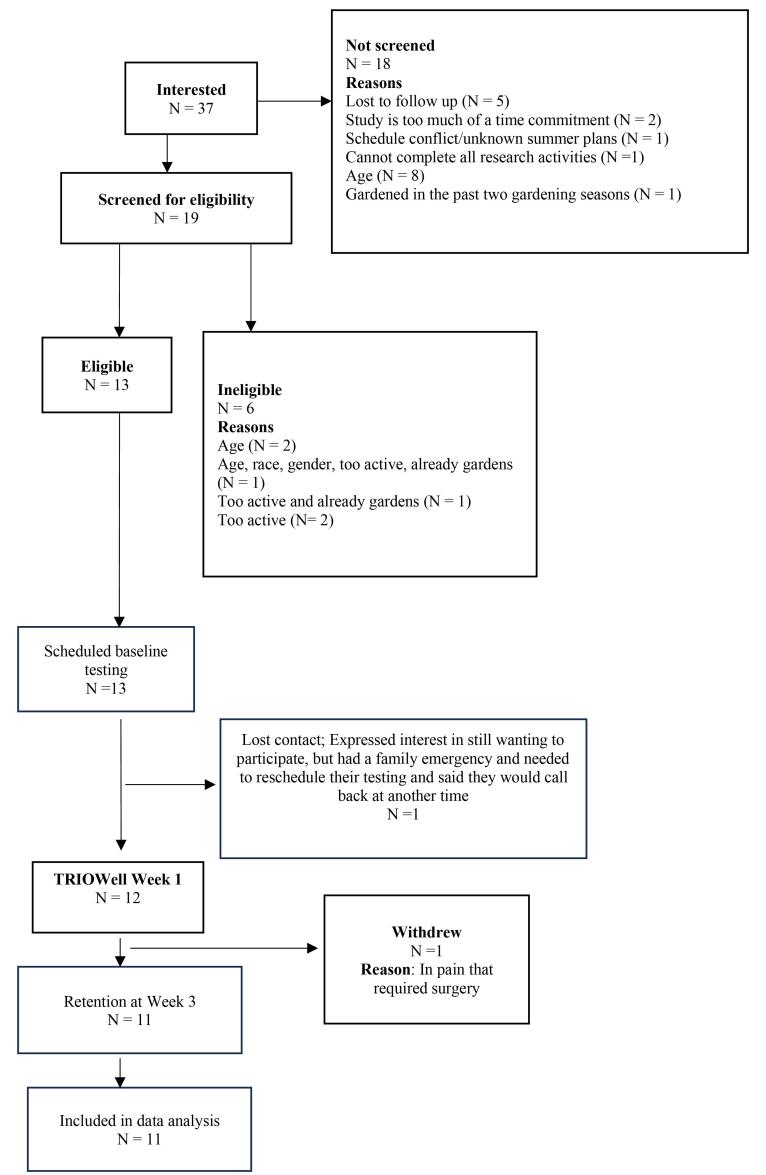
CONSORT (Consolidated Standards of Reporting Trials) Diagram Explaining Participant Flow

 Thirty-seven people expressed interest in participating in TRIOWell. Of these 37 people, 13 were eligible, and 12 provided informed consent to participate in the study, resulting in a 63% recruitment rate. Though, one participant withdrew from the study at week 3; this participant did not participate in any of the intervention due to increasing tooth pain and a change in her dental surgery date. Figure 1 provides a detailed participant flow.

####  Retention

 In total, 11 (100%) participants completed post-intervention surveys and interviews. Seven (63.6%) participants provided all post-intervention Fitbit data, surveys, and interviews.

####  Adherence

 On average, participants attended 60% (9/15 sessions) of the education workshops and 53% (8/15 sessions) of the gardening sessions. We also calculated attendance according to the day of the week. On average, participants attended 62.5% (5/8 sessions) of the Saturday education workshop sessions and 42.8% (3/7 sessions) of the Tuesday education workshop sessions. Participants attended 62.5% (5/8) of the Saturday gardening sessions and 57.1% (4/7) of the Tuesday gardening sessions.

####  Acceptability

 One item in the 19-item post-intervention evaluation asked, “Would you recommend this program to family/friends?” Of the participants who completed the evaluation (n = 9), 6 (66.7%) indicated that they would recommend this program to family and friends, 2 (22.2%) said that they were not sure, and 1 (11.1%) did not answer.

####  Additional feedback from participants about the feasibility and acceptability

 Among participants who completed the evaluations (n = 9), 7 (77.8%) indicated that they were satisfied with the education sessions and 2 (22.2%) indicated neutral. Of those who reported a neutral response, one person commented that “they were ok…I think I was expecting more ed. [education] related to actual gardening, nutrients, coaching.” All nine participants liked the gardening sessions and were satisfied with the historical connections in the education sessions. Seven participants (77.8%) felt that the gardening sessions encouraged them to be more physically active. One participant who was dissatisfied noted, “There weren’t any lessons or interactions to teach/show us how to bring the information into our daily routines.” Five participants (55.5%) reported satisfaction with the historical connections encouraging them to change their PA levels, 2 (22.2%) indicated that they were neutral, and 2 (22.2%) felt unsatisfied. Seven participants (77.8%) felt that Black History could be used in future health and wellness programs to help people be more physically active, while 2 (22.2%) felt neutral. One participant noted, “Yes, if the historical connections are physically active related; could consider adding info from black nutritionists, black trainers.” The feedback was quite extensive and largely indicated that more explicit explanations should have been made between the historical connections and gardening sessions, and how they were expected to translate into behavior change. However, many comments were also positive:

 “*The days I did attend was very educational, fun, learning about our historical connections to gardening, meeting new friends, and gardening itself was hard work, but fun at the same time. Will do it again.”*

 “*I realized that even the smallest movement was exercise and good for you.”*

 “*I used it as a reminder to keep up with my evening walks” *(Regarding the weekly phone calls/texts)

 “*It reaches back to our roots.” *(Regarding liking the gardening sessions)

 “*This should be taught as a course especially for youths” *(Regarding Black history being used for other health and wellness programming)

 “*Ms. Heather was great!” *(Regarding our horticultural educator)

###  PA Outcomes

 Wilcoxon analyses indicated an increase in daily step count from pre-to postintervention (baseline median = 4938l.91 steps per day; post-intervention median = 5819.38 steps per day; r = 0.53; *P*= 0.028). Effect sizes indicated increases in daily light PA (r = 0.45) and small increases in total daily PA (r = 0.29). There was also a small decrease in daily MVPA (r = -0.16). Analyses revealed a small increase in self-reported leisure PA (r = 0.17) and a small decrease in self-reported total PA (r = -0.17).

###  FV Outcomes

 FV intake had an 82-unit median score increase from 140 units at baseline to 222 units at 8-weeks (r = 0.51; *P =*.016). Effect sizes indicate that there was a small increase in self-reported green leafy vegetable consumption (r = 0.25). No pre-and postintervention changes were observed for self-reported consumption of other vegetables (r = 0, *P* = 1) nor berries (r = 0, *P* = 1).

###  Psychological health

 Effect sizes revealed a small decrease in perceived stress scores (r = -0.25) and a small increase in meaning and purpose (r = 0.15) from pre-to-postintervention. There was a small decrease in depression (r = 0.14) and self-efficacy (r = -0.35). There were no effects on anxiety or life satisfaction. Table 3 displays all PA, FV, and psychological health outcomes.

**Table 3:** PA, diet, and psychological health findings

**Variables**	**Baseline **		**Post-Intervention **		***P*** ** value**	**Effect size**^a^
	**Mean (SD)**	**Median (range)**	**Mean (SD)**	**Median (range)**		
**Fitbit Charge 3 PA (minutes per day) (n=7)**						
LPA	212.42 (63.70)	209.45 (149.10-274.55)	230.96 (65.97)	233.94 (173.75-290.33)	0.063	0.45
MVPA	7.35 (5.21)	8.20 (2.60-11.87)	5.74 (6.70)	4.75 (0-9.69)	0.499	-0.16
Total PA	219.77 (65.06)	213.65 (152.17-281.52)	236.70 (70.42)	243.63 (173.75-290.33)	0.237	0.29
Steps (per day)	5093.16 (1752.14)	4938.91 (3544.79-6369.21)	5897.71 (2347.18)	5819.38 (4177.25-6101.29)	0.028*	0.53
**Self-reported PA (MET-hours per week)**	Baseline (n = 9)		Post-Intervention (n = 11)			
Leisure	11.08 (8.54)	8.65 (3.8-16.82)	12.30 (9.94)	7.6 (4.85-18.10)	0.441	0.17
Work	34.55 (47.34)					
Total	45.62 (8.54)	43.19 (38.34-51.36)	46.85 (50.51)	42.14 (39.39-52.64)	0.441	0.17
**FV consumption (n=11)**						
Veggie meter (units)	182.51 (93.26)	140.33 (108.00-201.67)	251.85 (70.78)	222.33 (201.67-314.00)	0.016*	0.51
**Self-reported (MIND Diet Suboptimal Subsection Scores)**						
Green leafy vegetables	0.41 (0.38)	0.50 (0-0.50)	0.50 (0.32)	0.50 (0.50)	0.414	0.25
Other vegetables	0.36 (0.45)	0 (0-1)	0.36 (0.51)	0 (0-1)	1	0
Berries	0.50 (0.32)	0.50 (0.50)	0.50 (0.32)	0.50 (0.50)	1	0
**Psychological health (n=11)**						
PSS-10	15.55 (9.96)	16.00 (8.00-21.00)	13.73 (7.91)	16.00 (5.00-19.00)	0.342	-0.2
PROMIS anxiety	54.62 (2.59)	56.60 (47.00-60.80)	55.65 (2.35)	55.50 (52.00-58.40)	1	0
PROMIS depression	45.82 (3.72)	46.30 (38.20-51.60)	46.16 (3.42)	45.40 (38.20-51.70)	0.515	-0.14
BARSE	56.82 (24.36)	60.77 (33.85-75.77)	48.57 (22.31)	38.08 (33.92-75.38)	0.1	-0.35

^*^P < 0.05 ^a^r, effect size estimate. LPA, light physical activity; MVPA, moderate-to-vigorous physical activity.

## Discussion

 We hypothesized that a culturally tailored community gardening intervention would be a feasible approach to increase total daily PA levels, FV consumption, and psychological health among middle-aged AA women. Our hypothesis was partially supported where community gardening may be a feasible approach to increase PA levels and FV consumption among middle-aged AA women. However, the psychological health findings demonstrated mixed results, where stress, meaning and purpose and depression improved, anxiety and life satisfaction did not change, and self-efficacy decreased. Sixty-seven percent of participants (6/9) indicated that they would recommend this program to family or friends and post-intervention feedback from participants indicated generally enjoying the intervention but also provided suggestions of improvement in several areas. To the best of our knowledge, this is the first pilot community-gardening intervention that targets AA women, as well as the first PA-related intervention to include BHK as a mode of culturally-tailoring. Our findings are somewhat corroborated by other gardening studies, as well as by other culturally tailored PA interventions that target AA women.

 To date, one community gardening intervention has been published. Litt and colleagues^71^ conducted an observer-blind, randomized control trial (The Community Activation for Prevention Study [CAPS]) of a diverse adult population in terms of age, ethnicity, and socioeconomic status in a 1-year community gardening intervention and found that participants randomized to the community gardening group compared to the control group increased their fiber intake by 1.41 g per day and moderate-to-vigorous PA levels by 5.8 min per day, and saw greater reductions in perceived stress and anxiety in the intervention group. TRIOWell participants increased their median Veggie Meter assessed scores by 82-units [each 100 units of the Veggie Meter score corresponds to approximately one serving (cup) of FV consumed per day^72^] and reported a small increase in green leafy vegetable consumption. This finding suggests a potential increase in fiber intake amongst TRIOWell participants, given that FV contain fiber. FV consumption was specifically measured in CAPS, but there were no changes. Interestingly, TRIOWell nor CAPS directly targeted FV consumption, which may be why there were null findings for CAPS. It is plausible that the Veggie Meter scores (participants can view their scores during measurement and the scale is colorful) served as a form of self-monitoring, goal-setting, and self-regulation for TRIOWell participants, suggesting that interventions may not require direct diet counseling to elicit change in diet and that providing devices to self-monitor behavior may be adequate.

 In contrast to CAPS moderate-to-vigorous PA findings, moderate-to-vigorous PA levels in TRIOWell showed a small negative effect. During the intervention, we encouraged engagement in all domains of PA (i.e., transportation, household), many of which fall outside of moderate-to-vigorous PA. Participants were only engaging in 7-8 minutes of moderate-to-vigorous PA per day at baseline, so it may have required intentional programming with the goal of increasing moderate-to-vigorous PA engagement to see increases. Nonetheless, TRIOWell participants not only experienced a small increase in total daily PA, which supports our hypothesis, but they also increased their daily step count and had small increases in light PA. Participating in gardening 2-hours per week likely contributed to their increases in PA, but our emphasis on the benefits of participating in various forms and intensities of PA rather than focusing solely on the benefits of traditional forms of exercise may have translated to participants intentionally engaging in other forms of PA (i.e., transportation, household) outside of the intervention sessions. Our light PA findings are significant to note because of the recent interest in investigating light PA health benefits among adults.^73,74^ Furthermore, these findings suggest that non-traditional forms of movement may be efficacious approaches to increase PA among physically inactive populations. We also measured self-reported PA and participants reported small decreases in leisure PA, which also equated to small decreases in self-reported total PA. Given that self-report relies on recall and measures behavior, it is not uncommon for self-reported PA findings to differ from device-assessed PA findings, which measure movement.^75^ This finding underscores the need of including device-assessed measures of PA with self-reported measures.

 In both CAPS and TRIOWell, perceived stress decreased, however in our study we saw no change in anxiety whereas CAPS saw a decrease. Qualitative and observational studies have shown that gardening can reduce feelings of stress and anxiety amongst vulnerable populations.^76^ A meta-analysis of eight community gardening and horticultural studies^30^ overwhelmingly demonstrated positive associations between community gardening, well-being, and mental health outcomes, though one study within the meta-analysis found no difference between gardeners and non-gardeners self-reported symptoms of anxiety,^77^ suggesting that there may be other factors playing a role in the relationship between anxiety and community gardening. Additionally, CAPS was 1-year in duration and TRIOWell was an 8-week intervention, so TRIOWell may have been too short to elicit effects in anxiety.

 In contrast to CAPS, we also measured depression and markers of positive psychological well-being to further capture psychological health. A meta-analysis examining the effects of gardening on health^78^ demonstrated that gardening may reduce depression, which supports our depression findings. There may be a synergistic effect between PA engagement, being in nature, and social connection that contributes to reduced feelings of depression. More randomized control studies are needed to better understand which components of community gardening are specifically affecting health outcomes. To capture positive psychological well-being, we assessed meaning and purpose, general life satisfaction, and self-efficacy. In a qualitative study of community gardeners in New York City, the gardeners reported that gardening elicits a sense of purpose through their connections with nature and their community.^79^ TRIOWell participants experienced small increases in meaning and purpose. We measured meaning and purpose through a survey, so it is unknown exactly what caused the increase; however, we hypothesize that their reasons are like those expressed by the community gardeners in New York.

 We did not find a change in general life satisfaction. In a study examining the associations between time spent gardening and mental well-being and life satisfaction among middle-aged and older adults,^80^ spending at least 150 min/wk gardening was associated with higher general life satisfaction. Not only did TRIOWell participants garden for less than 150 min/wk (i.e., 120 minutes per week), but they also had a lower baseline general life satisfaction score compared to the general adult population. Middle-aged AA women may require extensive intervention to improve their general life satisfaction, so more research is necessary to better understand life satisfaction among this population. Our study also found that self-efficacy decreased. Self-efficacy in relation to gardening has largely been studied among children increasing their self-efficacy to increase their FV consumption,^81^ so less is known on the ability of participating in a community gardening intervention to increase self-efficacy to engage in PA behaviors when faced with barriers. Bandura^82^ posited that those who lack experience may have unrealistically high levels of self-efficacy for a specific behavior change. TRIOWell participants were low active and had little to no gardening experience, so this may explain their decrease in self-efficacy.

 Including BHK was a novel approach to culturally-tailor a PA intervention among middle-aged AA women, and it may have positively contributed to the findings, though future studies would need to refine some components. Traditionally, culturally-tailored PA interventions that have targeted AA women have included social support in the form of group-based PA and group discussion,^83,84^ faith-based/placed components, like interventions at the church and inclusion of prayer,^39^ and inclusion of electronic and mobile health.^84^ In a systematic review of 13 PA interventions for AA women, walking, social support, and healthy diet were significant strategies to promote PA in AA women, where seven of the studies increased PA among AA women and two demonstrated increases in FV consumption,^85^ which aligns with TRIOWell findings and underscores the importance of including components that are salient to AA women.

 Some women reported not seeing the connection between learning BHK and making health behavior changes. Suggestions to make the connection stronger included teaching about Black personal trainers or demonstrating exercises that correlated with movements specific to gardening. Additionally, the educational components could have focused on Black people’s historical contributions to gardening techniques and provided exercise demonstrations that build muscular and cardiovascular strength and endurance that is necessary for gardening. Despite the feedback, 100% (9/9; two women missed the final session when we gave out the evaluations) women said that they enjoyed the historical connections, 77.8% (7/9) felt that it could be used in future health and wellness programming to increase PA, and 66.7% of participants said that they would recommend this program to family/friends. This suggests that incorporating BHK has merit, but some changes should be made to its delivery before implementing another study.

 Adherence to the program was relatively low, which could suggest low feasibility, but holding intervention days on a Saturday yielded higher attendance than holding the intervention on a weekday and participants attended more gardening sessions than education sessions, suggesting that the participants enjoyed gardening. Additionally, the study took place during the summer, so one participant went on vacation for a few weeks during the study. Unfortunately, five women lost family members or friends during the duration of the study, one participant had a planned surgery, but suffered a stroke in response to the surgery, one participant got a new job, and it rearranged her schedule, one participant commuted 45-minutes to make it to the Saturday sessions, and two participants had inconsistent means of transportation. These factors contributed significantly to attendance, many of which were due to unforeseen circumstances. Despite these challenges, retention was at 63.6%, though 100% of participants completed the post-intervention surveys. This was largely due to research staff conducting the surveys face-to-face with the participants. Reasons participants did not provide post-intervention Fitbit data include no longer wanting to participate, the Fitbit causing a rash, and not liking biological data being tracked.

 Limitations of the study include the small sample size and lack of a control group. Additionally, the p-value could only be used as a reference point given the small sample size and non-parametric statistical methods used in the study. This limits the statistical implications of the study. However, the design of the study was appropriate for a feasibility study.^47^ Other limitations included inconsistent attendance Additionally, the length of the study was another limitation as 8-weeks may be too short to find meaningful changes in psychological health outcomes. Future PA studies may consider targeting a larger sample size, conducting a randomized controlled study, offering the program on the weekends only, and increasing the duration of the program to examine changes in psychological health outcomes.

 The present study had many strengths, including this being the first culturally tailored community gardening intervention targeting middle-aged AA women, as well as the first PA intervention to utilize BHK as a cultural tailoring method. Second, the study included a blend of device-assessed and self-reported PA and FV consumption measures. Third, the intervention leveraged existing green spaces and built connections with existing community organizations to implement the study. These strengths suggest several practical implications. First, the study offers a novel framework for designing interventions that resonate with AA women, utilizing cultural tailoring and behavioral theory to enhance the saliency^53^ and increase effectiveness.^86^ Second, optimizing and increasing the visibility of existing green spaces make interventions more cost-effective and scalable in community settings. Finally, collaborating with local community organizations fosters trust and supports the development of sustainable programs.

## Conclusion

 A culturally tailored community gardening intervention may increase PA and FV consumption, as well as improve some measures of psychological health among middle-aged AA women. Researchers may consider embedding BHK into other PA interventions to make it more culturally salient. However, adjustments should be made, including ensuring that there are explicit connections between BHK and PA behavior. Community gardening was acceptable to AA women, so future interventions should consider examining alternative approaches to traditional forms of exercise as a strategy to increase their PA levels. Lastly, community gardening as an intervention may be an effective approach to increase health equity because of its ability to target multiple levels of influence on healthy behaviors, such as providing local spaces to be active, providing opportunity to partner with existing community-based organizations, increasing food access and security, and increasing access to green, natural spaces.

## Competing Interests

 There are no competing interests to disclose.

## Ethical Approval

 The study was approved by The University of Illinois Urbana-Champaign Institutional Review Board. All participants provided written consent prior to data collection. Participants were free to withdraw at any point of the study. Research team members went over the potential risks and benefits of participation with the participants. Potential risks included being bitten by bugs, heat exhaustion, seasonal allergy exacerbation, muscle strain, Fitbit discomfort, and falls. Measures were taken to minimize these potential risks by helping participants adjust their Fitbits on their wrists during baseline testing, reminding participants to bend from their knees, and providing water. Potential benefits included health benefits from participating in gardening and findings from the study informing future studies.

## Supplementary Files

The following supplementary file is available: Supplementary file 1. Historical connections during the education workshops.
