# Supplementary material for: A culturally tailored community gardening approach to improving physical activity, fruit and vegetable consumption, and psychological health among African American women: A pre-post feasibility study
Source: Health Promot Perspect. 2025 Jul 15;15(2):122–37. doi: 10.34172/hpp.025.43243 (PMC12450582; doi:10.34172/hpp.025.43243)
Supplement: Supplementary file 1 — The following supplementary file is available: Supplementary file 1. Historical connections during the education workshops. [file hpp-15-122-s001.pdf]

Canton et al, **Health Promotion Perspectives.** 2025;15(2):122-S1
doi: 10.34172/hpp.025.43243
https://hpp.tbzmed.ac.ir

Supplemental Material

Table S1. Historical connections during the education workshops.

**Week**	**Topic**	**Historical Connection**
1.1	a. Getting to know each other<br>b. Introduction to Physical Activity	Anita J. Turner-first Black female physical educator in the US (1893)
1.2	Addressing Barriers to Physical Activity and Utilizing Physical Activity Assets	The "Black church" was the first institution built for and by Black people. It is a place for political, economic, spiritual, and social refuge<br><br>Can it also be used as a place to improve our health and increase PA?
2.1	Utilizing Community as Social Support to engage in healthy behaviors	Kinship networks arose out of response to enslavement; 18th and 19th century Black women made quilts to bond with each other, and quilting gave enslaved people time to socialize without supervision<br><br>How can kinship be applied to engaging in PA?
2.2	All about Community Gardening	"Handbook of the Negro Garden Club"- a book edited by Dr. H. Hamilton Williams for Black women to have at their garden clubs; "Gardening as Recreation" was a chapter discussing the health implications of gardening
3.1	History of African American Women and Gardening	*Entire workshop involves historical content*<br><br>Gardening was more than just gardening for many early 20th century African American women; it meant self-determination, community, and entrepreneurship
4.1	Food Justice/Power	In the Jim Crow era, land ownership was tied to voting rights, where African American farmers who voted, were

Week	Topic	Historical Connection
		evicted by white farmers, which left them with a choice between food/land or political rights
5.1	Food Systems	The Honorable Shirley Chisolm played a major role in the creation of Special Supplementation Nutrition Program for Women, Infants, and Children (WIC), which served to ensure that low-income women could have access to free, healthy food for their children
6.1	Climate Change and Health	Hazel Johnson founded the People for Community Recovery (1979) to address toxic industrial waste sites surrounding her Chicago neighborhood. She worked with a team to convince President Clinton to sign the Environmental Justice Executive Order (1994)
7.1	Environmental Justice	*Entire workshop involves historical content* North Carolina PCB (Polychlorinated biphenyls) Protest of 1982- the state dumped soil laced with PCB in Warren County, NC; residents (65% African American and 25% of residents lived in poverty) had no say in location of waste dumping sites, so they protested; The National Association for the Advancement of Colored People (NAACP) helped to organize protests that lasted for 6-weeks; it sparked new environmental justice movements led by poor and people of color communities
7.2	Environmental Activism throughout Black History	*Entire workshop involves historical content* “Jesus People Against Pollution” (1992) exposed a Columbia, Mississippi chemical company of their toxic dumping into African American communities; the dumpings resulted in health problems and increased mortality among Black people
